# What motivates audience comments on live streaming platforms?

**DOI:** 10.1371/journal.pone.0231255

**Published:** 2020-04-09

**Authors:** Mengdi Wang, Dong Li

**Affiliations:** School of Management, Harbin Institute of Technology, Harbin, China; University of Albany, State University of New York, UNITED STATES

## Abstract

This study aims to discuss audiences’ interaction and engagement with live streaming from a sociological perspective to investigate the different effects of information factors on audiences’ real-time interactions. Based on the interaction ritual chains theory, a data crawler was written using Python to collect data on the Huajiao platform for 1090 groups from 480 video game live rooms and 610 talent show live rooms. The results show that audiences’ commenting was mostly affected by the number of viewers, the gender of streamers, the number of likes, the number of gifts, and the duration of the live stream. Group comparison found that the effect of the number of viewers was significantly stronger in video game streaming, while the number of likes had a negative effect in video game streaming and a strong positive effect in live talent shows.

## Introduction

In recent years, social media have overwhelmed the Internet experience of users and academic researches. “Social media” refers to various online applications that enable the content created by an individual to be shared with others [1]. Social media are primarily intended to share diverse content and information via information systems [2]. No longer passive recipients of information, users now interact with other users online and make consumer-initiated contributions [3]. These forms of user interaction are known as user-generated content (UGC). This type of social media has a remarkable advantage of allowing users to collaborate easily and providing various ways to participate beyond the mere support of personal expression and communication [4]. This type of social media involves many new and varied platforms (e.g., YouTube, Facebook, and WeChat). Accordingly, the forms of computer-mediated communications have also developed rapidly. Text and image have shifted to audio and video. Users are also now inclined to interact in real time, compared with more conventional posts and reviews. Real-time video playing has emerged as a new form of social media that creates a special environment for streamers to share their shows and interact with audiences in real time using multiple media forms [5]. More specifically, streamers are able to upload their live data (e.g., video gaming and specialty show), or anything they want to perform. The audience in the same room can communicate with other audiences or streamers via bullet curtain (a specified name of a type of real-time comment) or send virtual gifts to the streamers they like.

Live video streaming has prospered since a growing number of people are immersed in this new type of social media. Diverse live streaming platforms also create a free and open environment for users to enjoy this type of interaction (e.g., Twitch and YouTube Live) [6].

Live video streaming has achieved remarkable progress in practice. However, it has received less attention in academic fields. Most studies of live video streaming have focused on the computer science and Information Systems realms (e.g., the optimization of live streaming systems), or examined scale and reliability problems [7–10]. Rarely have studies focused on explaining users’ emotional involvement or audiences’ continuous interactions. On the one hand, emotional involvement is closely associated with platforms’ capacity to foster a healthy connection with audiences, and it can cultivate firm adherence to viewers’ favorite platforms [11, 12]. On the other hand, user interaction significantly attracts and maintains users [5, 6].

However, no study has examined which specific information provided in a live streaming room generates the most real-time audience interactions. Some studies have analyzed the characteristics of the content and posts in knowledge sharing platforms or some professional groups based on linguistic theories [13–15]. In contrast to the previous focus on conventional social media or certain specific factors, this study aimed to gather all the basic information data showing in the live streaming room and investigate the different effects of these factors on audiences’ real-time interactions. Thus, this paper aims to discuss the audiences’ interaction engagement from a sociological perspective. Specifically, this paper will try to explain users’ interactive behavior in live streaming based on interaction ritual chain theory and statistically analyze the influence of different information on users’ interactions.

## Literature review and hypothesis development

### Interaction ritual chains

Collins proposed interaction ritual chains (IRC) theory as a combination of Durkheim’s concept of collective enthusiasm and collective signs and Goffman’s theory of situational small-scale study of human behavior [15, 16].

IRC consists of four basic elements (physical assembly by bodily presence, barriers to outsiders, common focus on an activity or an object, and shared common emotion or mood experience). Collins illustrated that the critical point represents a two-way recreation, and consciousness will generate a collective reaction of cognition. On that basis, there are four results of IRC (collective unity, spiritual power, representative signs of the bunch, and a sense of morality) [17]. Thus, IRC describes how emotional energy is generated through interaction rituals and also reveals the effects on a group’s solidarity and individuals’ participating states [18].

This theory perfectly interprets live streaming through certain theoretical mechanisms. First, audiences gathered in a common live room form a virtual co-presence with a barrier against outsiders. The streamer is the common focus of the audience and audiences can share the same emotion via real-time interactions (e.g., bullet curtains). As a result of the ritual, the streamer and audience build an impromptu community with high group solidarity and a sense of morality. Members in this group gain emotional energy as an independent entity, feeling confident, empowered, and passionate, and they initiate interactions. Also, symbols are generated based on those rituals (e.g., visual icons, bullet curtain, gifts).

A substantial amount of spiritual energy and two-way consciousness on a shared subject could improve participation, which is particularly true when people can play a crucial role in the interaction ritual instead of being auxiliary. Participation in rituals that allow individuals to be “performers” will lead to higher levels of emotional energy and greater involvement [19].

### Cognitive cost

Cognitive cost theory is vital to behavioral decision theory and creates a way to better understand consumers’ decision-making [20]. The cognitive cost theory suggests that customers weigh expenses and gains when making a decision [21]. The cognitive cost model theorizes that customers demonstrate restricted cognition and behavior when making decisions, which are determined by the relevant expenses of cognition [22]. Payne proposed a cost-benefit structure of cognition that has been endorsed by fellow scholars [23]. Based on this framework, consumers seek more details only when the benefit from further details exceeds the cost of doing so. Moreover, some researchers have introduced the concept of media richness to evaluate consumers’ cognitive cost. They suggest that medium or high media richness will be positively associated with complex decision-making [24]. In our study, various media forms in live streaming rooms can have different effects on audiences’ cognitive cost, so viewers will not participate in real-time interaction without considering the cognitive costs. Thus, those cognitive costs will to some extent affect the results of those consumer interactions in live streaming rooms.

## Hypothesis development

This study aims to explore consumer interactions in live streaming rooms in the form of real-time comment (bullet curtain) based on streamers’ information (e.g., level, gender, and number of fans), live timing information (e.g., starting time and length of time of the live stream), and audience information (e.g., the size of the audience, number of likes, number of gifts, and number of male and female viewers posting comment). This study also extends the types of live content category to video games and talent shows.

As mentioned above, live streaming fosters interaction ritual chains among the streamer and audience, and the audience generates a collective emotional effervescence based on a common focus or emotion. However, not all information in live streaming rooms has the same or equal effects on audience attention. Those live streaming platforms that provide particular communal circumstances exhibit an excessive capacity to influence respondents as they perform this interaction ritual chain based on anonymity, continuity, intimacy, and immediate accessibility [18]. Derived from this framework, our hypotheses are listed in Table 1.

**Table 1:** Summary of the hypotheses.

Variables	Benefits	Costs
**Level of streamer (H1)**	+	
**Number of fans (H2)**	+	
**Female streamer (H3)**	+	
**Starting time (H4)**		-
**Duration time of live (H5)**	+	
**Number of audiences (H6)**	+	
**Number of likes (H7)**	+	
**Number of gifts (H8)**	+	

Viewer information is critical in the conditions under which audiences initiate interactions. In accordance with Grice’s influential theory of conversation, the relevance of a conversation to group members may depend on who is the conversation starter [25]. Users act as selective disseminators, suggesting that they may consciously choose the object they communicate with [26]. Because people prefer contacting with “successful” people, a person with higher status (i.e., a higher level of streamers) (H1) or a person with more followers (i.e., the number of fans of the stream) (H2) is more likely to arouse others’ attentions in a group [27, 28]. Moreover, gender is also a vital influential factor on audiences’ participation. According to the fundamental assumption of social role theory, men show greater leadership (i.e., commanding and instruction, as well as competition) while women demonstrate collectiveness (e.g., friendliness, cooperation, and emotional expression) [29, 30]. More specifically, men are inclined to focus on the result, while women pay more attention to interpersonal relationships, i.e., a female streamer is more likely to arouse audiences’ emotional reactions and stimulate audiences’ interactions further (H3) [31].

The choice over time is significantly affected by the opportunity costs of watching and making comments on live streams. Such costs may impact audiences’ interactions differently. Some studies found users have round-number goal, and they prefer to purchase near a half-hour interval online (e.g., 19:28, 00:24) [32, 33]. Likewise, live streaming which start near round-number time may get more comments (H4). Moreover, the timing length of live streaming would have positive effect on users’ participation because it likely enhanced the viewers’ involvement [34]. Thus, the longer the streaming is live, the more comments they may get (H5).

IRC posits that feelings of effervescence will generate higher emotional energy via interaction ritual chains. Thus, the reactions of the whole group will impact the next effervescence more significantly [17]. In addition, from the perspective of customer behavior, information contributed by consumers is considered more trustworthy, and thus can raise other consumers’ interest [35, 36]. In other words, a viewer’s behavior in a live room will affect the other viewers. To be specific, the more viewers in the live room, the more comments will be received (H6). More likes (H7) and more gifts (H8) should increase the likelihood of audience commenting.

### Genres

Some researchers have proposed that what is contained in a video can affect how consumers behave when they view, endorse, and comment on it [37, 38]. Viewing patterns show distinct forms in different genres of videos [39]. On this basis, we suggest that audiences’ interaction styles will vary across current live video streaming contexts. As mentioned above, live streaming platforms create an open space for streamers to perform, and usually different channels are established based on different live contents. To be specific, video games and talent shows are the two commonest streaming genres.

In video game streaming, streamers share live video content of their ongoing games composited with a video feed of themselves. Streamers can chat with the audience and show their playing skills or experience simultaneously. Audiences in video games have more specific and centralized interests and knowledge of certain games than those in talent shows. Due to this similar interest and background, audiences in video game rooms are more likely to communicate actively and frequently.

Live streaming of talent shows is another major type of live streaming. Streamers in those channels primarily focus on various types of performance (e.g., singing, dancing, performing on instruments, or talk shows in real time). This type of live usually exhibits higher-level interactions among the streamer and audience members. The admiration of the viewers for the streamers will stimulate communication and consumption. Streamers will receive more gifts due their excellent performance or skills than in video game streaming.

## Methodology

Our methodology should fit the characteristics of our data. First, the quantity of real-time comments triggered in a live streaming room is a positive integer. Second, the quantity of real-time comments shows overdispersion (e.g., high variability and long tails) in different live streaming rooms. Third, in practice, the variance of real-time comments is several times the mean value of real-time comments. Thus, we adopt a count data model assuming the distribution of the underlying data to be Negative-Binomial [13].

### Model formulation

We assume that the number of real-time comments to *live stream i*, *Y*_*i*_,

$$P\left(Yi=k\right)=\frac{\Gamma \left(\lambda \text{i}/\vartheta +k\right)}{\Gamma \left(\lambda i/\vartheta \right)\Gamma \left(k+1\right)}\left(\frac{1}{1+\vartheta }\right)\frac{\lambda i}{\vartheta }{\left(\frac{\vartheta }{1+\vartheta }\right)}^{k},\;\lambda i>0,\vartheta >0,$$ (1)

where

*Y*_*i*_ is the number of real-time comments to *live stream i* (= 1,…, *N*), with *N* the number of live streams, and

*Γ* is the gamma distribution.

The Negative Binomial distribution is a two-parameter distribution. The two parameters are respectively *λ*_*i*_ and *θ*. The expected number of real-time comments to live stream *i*, E(*Y*_*i*_), is equal to *λ*_*i*_. The corresponding variance, Var(*Y*_*i*_), is equal to *λ*_*i*_(1 + *θ*). The parameter *θ* is often referred to as the overdispersion parameter. Larger values of *θ* represent greater overdispersion of the underlying data.

Next, we relate the lambda parameters to the explanatory model, and the model for the number of real-time comments (RECOM) to live stream *i* is:

$$\begin{array}{c} \text{log}\left(\lambda i\right)=\beta_{0}+\beta_{1}LEV_{i}+ln\beta_{2}FAN_{i}+\beta_{3}GENSTRE_{i}+\beta_{4}START_{i}\\ +\beta_{5}DURA_{i}+ln\beta_{6}AUD_{i}+ln\beta_{7}LIK_{i}+ln\beta_{8}GIF_{i} \end{array}$$ (2)

where

LEV_i_ refers to the level of streamers in live stream *i*;

lnFAN_i_ refers to the number of fans in live stream *i*;

GENSTRE_i_ refers to the gender of streamers in live stream *i*;

START_i_ 1 if the live stream *i* is start near round-number time, 0 otherwise;

DURA_i_ refers to the duration of live stream *i*;

lnAUD_i_ refers to the number of audiences in live stream *i*;

lnLIK_i_ refers to the number of likes in live stream *i*;

lnGIF_i_ refers to the number of gifts in live stream *i*;

### Data collection and sample

This study draws on data from two genres of live streaming (video game streaming and live talent shows). The data for these two types of live streams come from Huajiao, one of the most famous live platforms in China. According to an Aimei Report at the end of 2018, Huajiao live platforms had an average 23.9 million active users a month, higher than other platforms.

All the variables can be measured straightforwardly in the live room interface. Thus, a data crawler was written using Python according to the robots.txt to collect data on the Huajiao platform, and the data for 1090 groups were collected from 480 video game live rooms and 610 talent show live rooms.

For starting time, if the live stream starts near the round-number hours, we take it as “1” (e.g., 20:55–21:05; 01:25–01:35). Otherwise, we take it as “2” (e.g., 18:05–18:25; 18:35–18:55). For the duration of the live stream, we describe it in minutes and take every 30 min as 1 unit (e.g., if the live stream lasts for 26 min, “1” is taken; if it lasts for 45 min, “2” is taken). For streamers’ gender, we took male as “1” and female as “2.”

## Results

### Model estimation

Data analysis was conducted using STATA 14.1. Table 2 shows the descriptive statistics of the original variables investigated. According to the results of descriptive statistics, we found some types of data had a high mean and large span (e.g., fans, audiences, and gifts). This characteristic will impact the data analysis and may lead to small coefficients. Thus, to optimize the data analysis and narrow the data, the numbers of fans, audiences, likes, and gifts were converted to their logarithmic values for analysis. Table 3 lists the descriptive statistics for the logarithmic variables, and Table 4 the correlations among the logarithmic variables.

**Table 2:** Descriptive statistics of the original variables (N = 1090).

Variable	Mean	Std. Dev.	Min	Max
**RECOM**	673.33	936.47	12	2781.5
**LEV**	9.02	7.79	1	74
**FAN**	8709.05	15943.80	12	48826.5
**GENSTRE**	1.48	0.49	1	2
**START1**	0.37	0.48	0	1
**START2**	0.63	0.48	0	1
**DURA**	3.68	2.89	1	26
**AUD**	1630.07	2330.69	65	7251.5
**LIK**	687.74	627.43	37.5	1970.5
**GIF**	632525.80	1068036	2	3188272

**Table 3:** Descriptive statistics of the logarithmic variables (N = 1090).

Variable	Mean	Std. Dev.	Min	Max
**RECOM**	673.33	936.47	12	2781.5
**LEV**	9.02	7.79	1	74
**Ln FAN**	6.54	2.69	2.48	10.8
**GENSTRE**	1.48	0.49	1	2
**START1**	0.37	0.48	0	1
**START2**	0.63	0.48	0	1
**DURA**	3.68	2.89	1	26
**Ln AUD**	6.32	1.54	4.17	8.89
**Ln LIK**	5.93	1.27	3.62	7.59
**Ln GIF**	8.55	5.16	0.69	14.97

**Table 4:** Correlations among the logarithmic variables.

	**RECOM**	**LEV**	**Ln FAN**	**GENSTRE**	**START1**	**START2**	**DURA**	**Ln AUD**	**Ln LIK**	**Ln GIF**
**RECOM**	1.0000									
**LEV**	0.5313 ***	1.0000								
**Ln FAN**	0.6316 ***	0.8105 ***	1.0000							
**GENSTRE**	0.5041***	0.3483 ***	0.4877 ***	1.0000						
**START1**	0.0036	-0.0315	-0.0225	-0.0233	1.0000					
**START2**	-0.0036	0.0315	0.0225	0.0233	-1.0000	1.0000				
**DURA**	0.5053	0.3302***	0.3013***	0.2349***	-0.0003	0.0003	1.0000			
**Ln AUD**	0.8251 ***	0.5751***	0.6804 ***	0.5293 ***	-0.0122	0.0122	0.6471***	1.0000		
**Ln LIK**	0.6448***	0.4633***	0.4472***	0.3274***	-0.0132	0.0132	0.7722***	0.8410 ***	1.0000	
**ln GIF**	0.6412 ***	0.8014***	0.8910 ***	0.5525***	-0.0134	0.0134	0.3666 ***	0.7056 ***	0.5114 ***	1.0000

All *p*-values correspond to two-tailed tests of significance;

* *p* < .10;

** *p* < .05;

*** *p* < .01.

To judge whether there is an issue of multicollinearity, Variance Inflation Factors (VIFs) were calculated. VIF ranges from 1.00 to 6.62, and all indexes are below 10. Thus, as multicollinearity was unlikely to arise as a severe problem in this study, we proceeded to test the hypotheses.

### Estimation results

The Negative Binomial model of full sample is significant with *χ*^2^(8) = 3196.50, and *p* = 0.000. The Pseudo *R*^2^ of the number of real-time comments is 20.24%, indicating good fit of the model. Also, 29.08% and 9.06% of the variance was explained in video game and talent show live streaming, respectively. Table 5 shows the corresponding parameter estimates for this model.

**Table 5:** Parameter estimates for the Negative Binomial model for the full sample (*N* = 1091).

RECOM	Coef.	Std. Err.	Z-value	p-value
**LEV**	-0.0040	0.0031	-1.31	0.190
**lnFAN**	-0.0062	0.0132	-0.47	0.640
**GENSTRE**	0.1063***	**0.0348**	**3.06**	**0.002**
**START1**	0.0357	0.0283	1.26	0.207
**START2**	0 (omitted)			
**DURA**	-0.0173**	**0.0073**	**-2.38**	**0.017**
**lnAUD**	**0.6394** ***	**0.0244**	**26.17**	**0.000**
**lnLIK**	0.4706***	**0.0272**	**17.28**	**0.000**
**lnGIF**	0.0962***	**0.0068**	**14.18**	**0.000**

* *p* < .10;

** *p* < .05;

*** *p* < .01.

According to the factors in Table 5, three variables do not show significant effect on real-time comments: The level of the streamer, number of fans, and starting time. For the level of the streamer and the number of fans, we think this may be attributed to the frequent updates by the streamers. There are numerous new streamers appearing and old streamers disappearing from the platform every day. Audience are used to seeing new faces and are not obsessed with higher levels or more fans of various streamers. For starting time, we think that this may be attributed to the convenience of mobile devices. Users can watch live streams and post comments anytime they wish via the mobile equipment, so the starting time does not constitute a problem.

With respect to other variables, female streamers received more real-time comments than male streamers, supporting H3. However, contrary to our hypothesis, the duration of a live stream shows little negative effect on real-time comments (H4). Moreover, the numbers of viewers, likes, and gifts all show a positive effect on real-time comments, supporting H6, H7, and H8.

### Group comparison analysis

To verify the effect of live streaming genres, we conducted the subgroup comparison method according to Keil et al. [40]. Tables 6 and 7 depict the analysis results for video game and talent show streaming samples, respectively. To estimate the difference between two groups, we performed seemingly unrelated regression (SUR) using STATA, followed by the sub-group coefficient test. The *χ*^2^ and *p*-values of different variables are listed in Table 8.

**Table 6:** Parameter estimates for the Negative Binomial model for game videos (*N* = 480).

RECOM	Coef.	Std. Err.	z	P>|z|
**LEV**	-0.0094**	**0.0041**	**-2.32**	**0.021**
**Ln FAN**	-0.0208*	**0.0114**	**-1.81**	**0.069**
**GENSTRE**	0.0464	0.0295	1.57	0.116
**START1**	0.0151	0.0239	0.63	0.527
**START2**	0 (omitted)			
**DURA**	-0.0361***	**0.0071**	**-5.10**	**0.000**
**Ln AUD**	1.4751***	**0.0709**	**20.82**	**0.000**
**Ln LIK**	-0.1969***	**0.0475**	**-4.14**	**0.000**
**Ln GIF**	0.0389***	**0.0056**	**6.95**	**0.000**

* *p* < .10;

** *p* < .05;

*** *p* < .01.

**Table 7:** Parameter estimates for the Negative Binomial model for talent shows (*N* = 610).

RECOM	Coef.	Std. Err.	z	P>|z|
**LEV**	-0.0006	0.0046	-0.13	0.893
**Ln FAN**	-0.0231	0.0214	-1.08	0.280
**GENSTRE**	-0.1352**	**0.0615**	**-2.20**	**0.028**
**START1**	0.0159	0.0438	-0.36	0.716
**START2**	0 (omitted)			
**DURA**	-0.0330***	**0.0124**	**-2.67**	**0.008**
**Ln AUD**	0.4898***	**0.0293**	**16.74**	**0.000**
**Ln LIK**	0.7027***	**0.0433**	**16.22**	**0.000**
**Ln GIF**	0.0651***	**0.0240**	**2.71**	**0.007**

* *p* < .10;

** *p* < .05;

*** *p* < .01.

**Table 8:** Sub-group coefficient test.

Variables	χ^2^	p-value
**LEV**	2.10	0.1475
**Ln FAN**	0.01	0.9289
**GENSTRE**	3.81	0.051
**START1**	0.00	0.9881
**START2**		
**DURA**	0.03	0.8554
**Ln AUD**	**40.78**	0.0000***
**Ln LIK**	**62.41**	0.0000***
**Ln GIF**	1.47	0.2259

* *p* < .10;

** *p* < .05;

*** *p* < .01.

Consistent with the results for the full sample, the duration time has negative effect and the number of gifts has positive effect in both genres. And the level of streamer and the number of fans show negative effect on real-time comments in game video streaming while the male streamers shows positive effect on real-time comments in talent show streaming.

According to the two sub-groups comparison, the positive effect of the number of audiences on real-time comments is greater for game video streaming than talent show streaming. In addition, the number of like in talent show seems to be a strong stimulation on real-time comment rather than negative influence in game videos.

## Implications

### Theoretical implications

This study sought to investigate consumer engagement in live streaming viewed from sociological and participation perspectives. For this study’s theoretical contribution, we focus on the interactive framework. This study provides insight into how interaction ritual information affects users’ interaction.

First, live streaming has not received sufficient attention in the academic realm; only a few studies have explored the operation of live streaming systems. As far as we are aware, this is a pioneering study of the audiences’ real-time interactive conduct in live streaming in terms of social psychology and communication perspectives. More specifically, this paper explains the mechanism of the interaction of the streamer and audience in a live streaming room in accordance with interaction ritual chains theory (IRC). Our explanation of real-time users’ interaction in such an impromptu community incorporates communication theory into this new type of real-time social media platform.

Second, our study illuminates the users’ interaction mechanism in real-time interaction on social media. Some research has concentrated on exploring the impact factors of regular comments or reviews in traditional social media, such as Twitter and Facebook. Several papers have focused on how features and characteristics of a post drive the number of comments it receives on some UGC forums from a semantic perspective. Recently, some studies have verified the users’ continuous intention of live streaming from the perspective of social identification. Based on this research, we sought to further examine users’ interaction triggers on live streaming. Thus, we performed an experiment to analyze the characteristics that impact the number of real-time comments that a streamer receives in one live streaming room. Data from live streaming rooms were collected from one of the most famous live streaming platforms in China, Huajiao. The features of streamers’ level, streamers’ gender, starting time, duration of live streaming, and numbers of fans, audiences, likes, and gifts were verified in this study. The test findings suggest that (1) female streamers have greater advantages than male streamers, (2) overlong show times will negatively affect the number of real-time comments, and (3) the numbers of viewers, likes, and gifts could contribute to the number of real-time comments. Thus, this study constitutes a profound analysis of data on how users of the live streaming behave.

Third, two major genres of live were compared and analyzed. First, the effect of those characteristics is examined in video game and talent show live streams. Most hypotheses received similar results to those of the full sample. Second, the two groups were compared based on sub-group coefficient test to show that male streamers have an advantage in game video streaming, females in talent show streaming. For the number of viewers, audiences of video game streaming made more contributions than those of talent shows. This finding implies that different genres of live streaming have different patterns of user behavior that may impact audience interaction styles, suggesting that platforms can implement different and customized strategies in certain streaming types.

### Practical implications

This study suggests implications of the new types of social media platforms from the perspective of marketing communication, especially regarding how online social media communities can gain more user-to-user interaction.

First, this study extends our understanding of the emerging media through research on connections and high user-interaction platforms [41]. Live streaming platforms combine various media types (e.g., video, text, and picture) and different participants (e.g., audiences, streamers, and platforms). Given their rapid development and competition, the operators of live streaming platforms should understand the inner mechanism of users’ participating characteristics. This study creates a novel way to interpreting the activities in live streaming based on interaction ritual chains theory (IRC). The interaction ritual chains join all the behavior nodes in live streaming through symbols and emotional energy, implying that platforms can stimulate users’ behavior by controlling specific symbols.

Second, live streaming platform operators should consider the features that impact users’ participating behavior effectively. UGCs are usually considered indicators of consumer activities that could be used to predict users’ interaction. However, treating these interactions as homogeneous does not take the observed behavioral differences into account. Also, different information indicators in live streaming are analyzed, and only several specific influencing factors were found. On the one hand, managers in live streaming should control for an appropriate gender ratio since female streamers are more attractive than male streamers. On the other hand, users’ individual behavior (e.g., likes and gifts), i.e., user-streamer interaction, can stimulate user-user interaction.

In addition, the comparative analysis of two major types of live streaming revealed that platform managers should differentiate the operation and marketing of different genres of live streaming. For instance, platforms can promote more female streamers in live talent shows or introduce targeted marketing on different channels. Customized strategies can be implemented in different genres of stream lives. For talent show live streams, platforms could provide some competitions (e.g., beauty contests or star competitions). Also, gathering “likes” can be set up as an activity to help streamers enhance their competitiveness.

### Limitations and subsequent research

First, our results are limited by the fact that the data were collected from one Chinese live streaming platform. Future studies should extend the scope of data to other platforms.

Second, all the data we collected are direct and visual. However, there is other critical information in live streaming rooms (e.g., the content of comments) that contains indications of users’ attention and other preferences. Thus, in future studies, appropriate methods should be employed to obtain and process these types of data.

Third, only two major genres of live streaming (video game and talent show streaming) were compared. In fact, many other types are available to audiences (e.g., sports and education). Also, how consumers behave might differ. Thus, future research ought to integrate diverse playing types and perform specific comparative studies to better comprehend the effect of what is streamed.

## Supporting information

S1 Data (XLSX)
